# Worldmapper: The Human Anatomy of a Small Planet

**DOI:** 10.1371/journal.pmed.0040001

**Published:** 2007-01-30

**Authors:** Danny Dorling

## Abstract

The Worldmapper Web site is a collection of world maps where territories are re-sized according to the subject of interest.

## The Challenge: Understanding Global Inequalities

*“Throughout the world, people who are vulnerable and socially disadvantaged have less access to health resources, get sicker, and die earlier than people in more privileged social positions. Health equity gaps are growing today, despite unprecedented global wealth and technological progress” [1].*

You can say it [1], you can prove it [2], you can tabulate it [3], but it is only when you show it that it hits home [4]. There is a long history of using illustrations to help spread new medical scientific ideas. Anatomical imagery, for example, is at least 500 years old [5]. That imagery allowed us to look inside human beings and, among much else, showed us just how much of the brain was dedicated to visual understanding.

We now know that good health relies as much on the anatomy of society as on the anatomy of our bodies [6]. And we are just beginning to learn that an unequal human world is also more likely to be a sick world. How, though, can we better understand the distribution of health resources around the world, and of where most people are sick and die early as compared to people in more privileged positions? How can we fathom the extent to which health equity gaps are growing despite unprecedented global wealth and technological progress? Drawing images is one way to engage more of our imagination to help understand the extent and arrangement of world inequalities in health.

## Medical Mapping

The science of the make up of world human anatomy—cartography—has a similar history to that of anatomical drawing. Gerardus Mercator's wall maps of 1569 were produced just 14 years after the second edition of Andreas Vesalius' *humani corporis fabrica*. And just as Vesalius' images helped guide the scalpel through flesh, Mercator's maps helped guide ships across the oceans. But these products of the enlightenment were not just simple guides. The images they produced helped change the way we thought about the world. In the long run they helped us learn to be less superstitious, but also presented a very mechanical, inhuman image of both person and planet.

Mercator's projection is the one you still see when the weather is described on television and it, or a near equivalent, is the one used in most medical mapping (for an example depicting the world geography of malaria see [7]). The Mercator projection is a useful projection to carry with you if you wish to sail around the planet. It is not, however, that useful for showing how a disease such as malaria is spread amongst the population. To show such spread, a world population cartogram is a far better base map—an example is shown in [8].

The Mercator projection is the worst of all the well known global map projections to use to depict disease distributions because it stretches the earth's surface to the most extreme of extents and hence introduces the greatest visual bias. On a Mercator projection area is drawn in ever expanding proportion to how near territory is to the poles. Thus, on such a projection, India appears much smaller than Greenland, whereas India is in land area alone over seven times larger than Greenland. The world distribution of malaria shown on a conventional map [7] gives the impression that the global distribution of clinical episodes of Plasmodium falciparum malaria is confined to a much smaller proportion of the earth's surface than is actually the case.

However, even if the distribution of a disease such as malaria had been drawn on an equal land area map it would still give the impression that malaria was only confined to a small portion of the world's land. Malaria is, however, a disease of people, not of land. A better base-map (had it been available) upon which the distribution could have been drawn would have been the population cartogram [8]. My aim here is not to specifically criticise the depiction of the distribution of malaria on a conventional map. Such depiction is simply representative of what is accepted as normal in much medical mapping, even by authors with access to software that allows them to produce non-unique cartograms [9].

## The Solution: Creating Maps of Inequalities

The new world population cartogram published in *Nature* in 2006 [8] was produced by Mark Newman [10] and shows the world with land area drawn *in proportion to the population*. Unlike previous cartograms, this cartogram is produced by software which approximates to the best unique world cartogram (that which distorts the least on the surface of the sphere whilst still scaling areas correctly). One criticism of older algorithms has been that they produce an “area correct” but somewhat arbitrary solution with an end result that often reflects the initial projection used. For any given distribution there are an infinite number of cartograms that can be drawn with area in proportion to that distribution, but only one which also minimises distortion. That first shown in *Nature* [8] is just such a cartogram. Put most simply, if rates of malaria were drawn upon the equal population map, then the area shaded as being affected by malaria on that map would at least relate to the number of people living at risk of malaria—and not to land area.

Newman's new projection required the adaptation of a previously working computer algorithm to allow it to produce a cartogram on the surface of the sphere. Once adapted for world mapping the algorithm could be used to show much more than simply population distribution. Furthermore, unlike its predecessor projections, Newman's does not reflect the arbitrary choice of initial projection [11] (for instance it joins East–West unlike any other equal population projection). Newman's algorithm produces an image that approximates a unique least distorting solution. This means that the reader has only one new projection to learn should they wish to map upon population rather than land. But for the more imaginative of readers, why just stop at one?

The human eye–brain system is far better equipped to judge the relative values of objects by the size of the area that each object occupies rather than to translate shades of colour into rates and then imagine what they imply. An example of the effectiveness of rescaling area to show value from medical illustrations is the traditional homunculus upon which the skin of a human is re-scaled in proportion to even out number of nerve endings. See, for example, http://en.wikipedia.org/wiki/Homunculus; what you see is that our hands and lips are large, and our genitals are much smaller, in terms of sensitivity, than many of us may have imagined.

**Figure 1:**
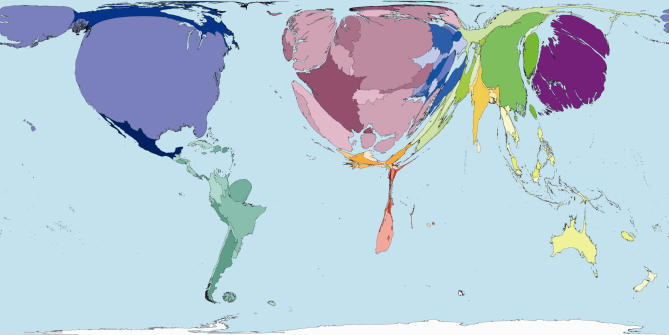
Public Health Spending: Worldmapper Poster 213 The figure shows a cartogram in which territories are drawn with their area in proportion to the values being mapped. Territories are shaded identically on all cartograms here to aid comparison with the world cartograms shown in Figures 2 to 6 which employ identical shading. For detail on shading see http://www.worldmapper.org. Source of data used to create map: United Nations Development Programme, Human Development Report 2004.

**Figure 2:**
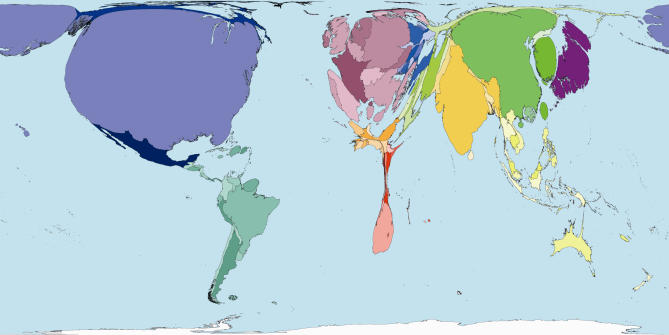
Private Health Spending: Worldmapper Poster 214 Source of data used to create map: United Nations Development Programme, Human Development Report 2004.

**Figure 3:**
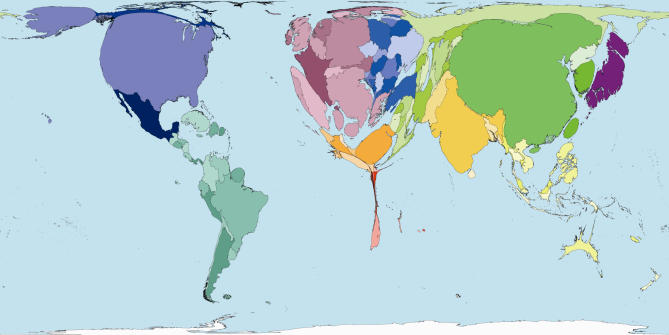
Physicians Working: Worldmapper Poster 219 Source of data used to create map: World Health Organization, 2004, Human Resources for Health, Basic data.

**Figure 4:**
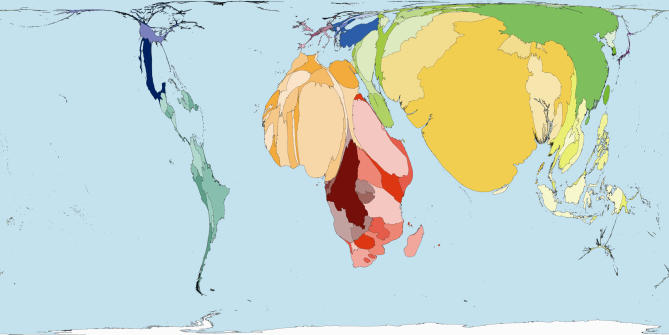
Early Neonatal Mortality: Worldmapper Poster 260 Source of data used to create map: World Health Organization, 2005, World Health Report, Basic data.

**Figure 5:**
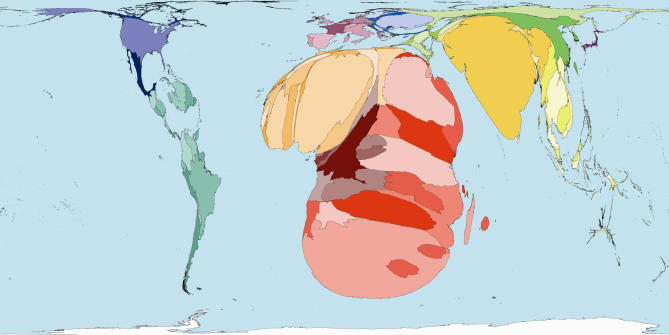
HIV/AIDS Prevalence: Worldmapper Poster 227 Source of data used to create map: United Nations Development Programme, Human Development Report 2004.

**Figure 6:**
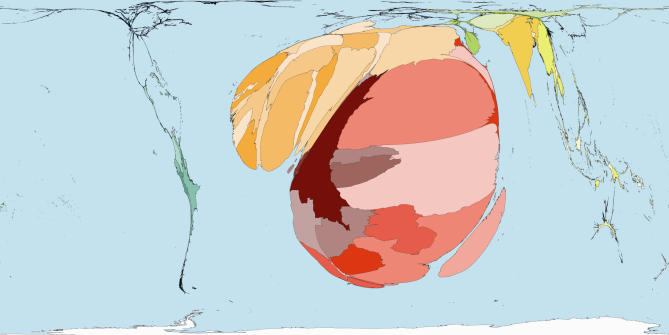
Malaria Cases: Worldmapper Poster 229 Source of data used to create map: World Health Organization and UNICEF, World Malaria Report 2005.

The homunculus presents an image that is not easy to forget. In that tradition then, consider some maps of the world rescaled to show the areas of territories drawn in proportion to the amounts of monies spent publicly and privately on health (Figures 1 and 2), and drawn in proportion to the number of working medical staff available to treat the sick in each place (Figure 3). Or consider the world rescaled to show the almost wholly avoidable annual deaths of 3 million infants in their first week of life (Figure 4), the millions currently living with HIV/AIDS (Figure 5), of whom 3 million died last year, and the tens upon tens of millions of people who experience malaria each year (Figure 6).

Details of the sources of data for these maps are given in brief beneath each figure, and in greater detail on the Worldmapper Web site (http://www.worldmapper.org). Note, however, that the choice of map projection and even of specific cartogram algorithm usually has a far greater effect on what you see than most ambiguities over data estimation. In the case of malaria especially, different estimates of risk (rather than the number of cases) produce very different images [12].

Worldmapper is a collaboration between researchers at the Social and Spatial Inequalities Research Group of the University of Sheffield, United Kingdom, and Mark Newman, from the Center for the Study of Complex Systems at the University of Michigan in the United States. During the course of 2006, the project aimed to create 365 new world maps, embed them in explanatory posters, and provide raw data and technical notes on many of the most prominent of the world major datasets published mainly by various United Nations organisations. This information is all provided open-access through the Web site. Only a minority of the maps concern issues of medical services, diseases, and ill health—but these are related to almost everything else that is mapped. By the end of October 2006, over 893,000 hits were already recorded on the then far from complete Web site.

As a species we are just beginning to learn that unprecedented global wealth and remarkable technological progress does not result in the reduction of inequalities in health, nor in a decline in human suffering, nor in an enlightened and glorious new age. An anatomically correct technical image of the planet or the human body is not enough for us to learn how to better live with each other, nor to see other individuals or distant large groups of people as human. But new ways of depicting the world and people can change how both it and we are seen; possibly for the better. Traditional illustrative anatomy, just like scientific cartography, can de-humanise. The human body was first clearly laid bare in ink for the beginnings of modern surgery in 1543 [5], and the coastlines of the continents were first consistently exposed on paper for crude mercantile trade in 1569 [11]. For almost all of the last five centuries these “scientific” human and world views have shaped our thinking in one particular direction. They have suggested purely technical fixes, implied we were enlightened, and reinforced a world view that progress simply requires more detailed understanding rather than re-thinking, new imagination, and greatly expanded empathy. The maps in Worldmapper are part of a much wider attempt to see and think differently.

## Next Steps

Currently the maps in the Worldmapper project are two-dimensional and are not particularly interactive. It could be possible to spin them around the sphere and allow viewers to zoom in and out of the globe and query where they were looking—to find out more about each place, to learn more, more quickly, and even to see one image morph into another. We are hoping to create such 3-D images, and by the time you read this article, the Worldmapper Web site may well have such spinning globes.

We also hope to create further global health maps early in 2007 beyond those already shown in the 365 created in 2006. One plan we have is to produce over 100 extra maps of all major causes of death based on new estimates for the year 2002 recently released by the World Health Organization. We aim to work on these 100 maps in spring 2007, after the first 365 new world maps have all been put on the web. Worldmapper maps 368 to 484 will show the global distribution of all major world diseases, self-inflicted deaths, and aggregations of the two. Further, it is planned that maps 485 to 528 will show images of the world re-sized according to the numbers of people who die for subgroups of both sexes in 22 age groups. Finally, in this new section of the Worldmapper site, map 367 will show what a more equal world would look like by sizing territory in proportion to the numbers that would be expected to die in each territory were mortality rates by age and sex equal worldwide. When map 367 is seen and compared to map 368—of the numbers observed that actually die in each place in each year—the extent of world health inequalities will hopefully be made clearer still. In contrasting these two images, those inequities masked by the much younger age profile of the poorer majority of the world's population will be revealed.

One of many limitations to the project is the fact that currently only 200 territories are mapped by Worldmapper. These are the member states of the United Nations and a few dozen other territories and areas that contain many people, or that cover a large area, and for which values are sometimes provided by international organisations, or can be estimated. Incidentally, trying to estimate the number of countries in the world's human anatomy is rather like trying to count the number of bones within a single human body—the number varies depending on your point of view and the age of the subject.

Looking further into the human anatomy of this small planet, in addition to adding more subjects to map, we could begin to think of how to fly in, to look at variation within the borders of the state as well as between territories, and to move beyond the nation-state as our means of categorising people. There is much more that could be done. However, what I think matters most are the new ways of thinking that we foster as we redraw the images of the human anatomy of our planet in these ways. What do we need to be able to see—so that we can act?
